# PP31 Patient And Citizen Involvement In Decision-Making About Health Technologies: The Case Of Button Feeding Tubes In Brazil

**DOI:** 10.1017/S026646232510250X

**Published:** 2025-12-29

**Authors:** Milene Rangel da Costa, Bruno Barros, Quenia Cristina Dias Morais, Marisa Santos, Andressa Braga

## Abstract

**Introduction:**

Patient and citizen perspectives are a cornerstone of health technology assessments (HTA), ensuring transparency and alignment with societal needs. This study examined the role of public opinion on the evaluation of button feeding tubes for recommendation in Brazil’s Unified Health System (SUS). The objective was to assess how public opinion shaped the final recommendation ensuring equitable access to this technology.

**Methods:**

This was a qualitative review of the contributions received by the Brazilian Ministry of Health during the public consultation process about button feeding tubes in 2021. Data sources included contributions from various stakeholders, which were analyzed for their alignment with the preliminary recommendation and their impact on the final deliberation.

**Results:**

A total of 425 contributions from patients, caregivers, healthcare professionals, and industry representatives were analyzed. All of them supported the recommendation of button feeding tubes for pediatric patients in need of long-term enteral nutrition. Overall, they highlighted the advantages of the technology compared to long gastrostomy tubes such as reduced complications, cost effectiveness, ease of use, and improved quality of life. Their impact on patient dignity and social inclusion was also mentioned. Transparent communication throughout the process fostered trust and encouraged meaningful participation. Stakeholder input significantly influenced decision-making, particularly in prioritizing equitable access and designing implementation strategies.

**Conclusions:**

Patient and citizen involvement proved crucial to the unanimous agreement to recommend the inclusion of button feeding tubes in the SUS. Their perspective on the technology’s potential to improve patient care and resource allocation was an important component to decision-making. This case highlighted the value of public engagement in fostering transparent and equitable HTA processes.

